# Phytotoxic Activity and Chemical Composition of Aqueous Volatile Fractions from *Eucalyptus* Species

**DOI:** 10.1371/journal.pone.0093189

**Published:** 2014-03-28

**Authors:** Jinbiao Zhang, Min An, Hanwen Wu, De Li Liu, Rex Stanton

**Affiliations:** 1 Key Laboratory of Biopesticide and Chemical Biology, Ministry of Education, and School of Life Sciences, Fujian Agriculture and Forestry University, Fuzhou, China; 2 Environmental and Analytical Laboratories, Faculty of Science, Charles Sturt University, Wagga Wagga, New South Wales, Australia; 3 E.H. Graham Centre for Agricultural Innovation (a collaborative alliance between Charles Sturt University and Industry & Investment NSW), Wagga Wagga Agricultural Institute, Wagga Wagga, New South Wales, Australia; Islamic Azad University-Mashhad Branch, Iran (Islamic Republic of)

## Abstract

The essential oils from four *Eucalyptus* species (*E. spathulata*, *E. salubris*, *E. brockwayii* and *E. dundasii*) have been previously confirmed to have stronger inhibitory effects on germination and seedling growth of silverleaf nightshade (*Solanum elaeagnifolium* Cav.). The aqueous volatile fractions (AVFs) were the water soluble volatile fractions produced together with the essential oils (water insoluble fractions) during the steam distillation process. The aim of this study was to further assess the phytotoxicity of AVFs from the four *Eucalyptus* species and their chemical composition. The fresh leaves of the four *Eucalyptus* species were used for the extraction of AVFs. The AVFs were tested for their phytotoxic effects on the perennial weed, silverleaf nightshade under laboratory conditions. The chemical compositions of the AVFs were determined by gas chromatograph–mass spectrometry (GC-MS). Our results showed that the AVFs had strong inhibition on the germination and seedling growth of silverleaf nightshade. The inhibition index increased with the increasing concentrations of AVFs. The inhibitory effects of the AVFs varied between different *Eucalyptus* species. The AVF from *E. salubris* demonstrated the highest inhibitory activity on the weed tested, with complete inhibition on germination and seedling growth at a concentration of 75%. The GC-MS analysis revealed that 1,8-cineole, isopentyl isovalerate, isomenthol, pinocarvone, *trans*-pinocarveol, *alpha*-terpineol and globulol were the main compounds in the AVFs. These results indicated that all AVFs tested had differential inhibition on the germination and seedling growth of silverleaf nightshade, which could be due to the joint effects of compounds present in the AVFs as these compounds were present in different quantities and ratio between *Eucalyptus* species.

## Introduction

Eucalyptus has been reported to have a range of bioactivity, including antimicrobial, antiviral, fungicidal, insecticidal, anti-inflammatory, anti-nociceptive and anti-oxidant activities [1]–[3]. It was also reported that essential oil from eucalyptus has strong phytotoxic effects against many weeds, such as *Parthenium hysterophorus*, *Cassia occidentalis*, *Echinochloa crus-galli*, *Bidens pilosa*, *Amaranthus viridis*, *Rumex nepalensis*, *Leucaena leucocephala*, *Casuarina pusilla* and *Leptospermum myrsinoides* [4]–[7].

Similarly, aqueous extracts or leachates of eucalyptus have also been documented to possess bioactivities [8]–[11]. Aqueous leachates from fresh leaves of *Eucalyptus globulus* Labill. were suppressive to the establishment of vegetative propagules and early seedling growth of purple nutsdge (*Cyperus rotundus* L.) and bermuda grass (*Cynodon dactylon* L.) [8]. Khan et al. [9] reported that aqueous extracts from *E. camaldulensis* L. significantly inhibited weed germination and seedling growth. The water soluble fractions of *Eucalyptus dundasii* obtained during steam distillation were reported to be phytotoxic to the germination and growth of annual ryegrass (*Lolium rigidum* Gaudin) and barley grass (*Hordeum glaucum* Steud.) [11]. The phytotoxic activity of eucalyptus extracts obtained by different methods suggests that they may have potential herbicidal activities.

Field observations have identified that there was limited vegetation within the dripline of four *Eucalyptus* species: *E. spathulata*, *E. salubris*, *E. brockwayii* and *E. dundasii*. The presence of these *Eucalyptus* species also suppressed the understorey growth of silverleaf nightshade (*Solanum elaeagnifolium* Cav.).

Silverleaf nightshade is a deep-rooted, summer-growing perennial weed of the Solanaceae family that is a declared noxious weed in several countries [12]–[13]. It has been recently listed as one of the Weeds of National Significance in Australia [14]. The management of this weed includes cultural, mechanical, chemical and biological controls [12]. In the absence of reliable and effective control options, alternative control options are needed for the effective management of this weed.

Our previous study has confirmed that the phytotoxicity of essential oils (water insoluble fractions) from leaves of these four *Eucalyptus* species on silverleaf nightshade [15]. The essential oils were extracted by steam distillation from the leaves of the four *Eucalyptuses*. Meanwhile, an aqueous volatile fraction (AVF) was also obtained during the steam distillation. The aim of this study was to further assess the phytotoxicity of AVFs from the four *Eucalyptus* species and their chemical composition.

## Materials and Methods

### Plant Materials and Chemicals

Approximately two kilograms of fresh leaves of *E. spathulata*, *E. salubris*, *E. brockwayii* and *E. dundasii* were randomly collected from 6-year old trees grown in the field at Ungarie (Long. 146°55′41.33″, Lat. 33°35′53.06″), New South Wales (NSW), Australia. *E. melliodora* was included as a control species as it had no suppression on the understory vegetation as compared with above four *Eucalyptus* species underneath which silverleaf nightshade and other vegetation were suppressed. The fresh leaves of *E. melliodora* were collected from Wagga Wagga campus, Charles Sturt University and used as a control species. The leaves were then stored in a cool room (10°C) before steam distillation. Seeds of silverleaf nightshade were collected from a field site at Culcairn (Long. 147°10′7.75″, Lat. 35°35′38.11″), NSW in 2008. No specific permissions were required for these locations/activities because this was done on public area. The field studies did not involve endangered or protected species.

### Steam Distillation

AVFs were extracted by steam-distillation according to Wu et al. [11]. Three hundred grams of fresh leaves of eucalyptus leaves were cut into 5 mm strips and subjected to steam-distillation for 2.5 h using a Pyrex oil distillation apparatus with a flat bottom flask (2 L) containing 1,200 ml distilled water to generate steam. The volatile components from the leaves were condensed through a cooling tube. Two volatile fractions, which included condensed water and the fractions (defined as “essential oil”) afloat on it, were obtained. The former was collected through a separation funnel and designated as the AVF (full strength, 100%), which was stored in a sealed bottle at 4°C before use.

### Bioassays of AVFs on Weed Germination and Growth

The bioassay protocol developed by Wu et al. [16] was adopted. A concentration series [0 (water control), 25, 50, 75, 100%] was made up from the full strength (100%) solutions of AVF. Seeds of silverleaf nightshade were pre-treated by soaking in water for 24 h to remove the mucous coating for improved germination [17], then air-dried and stored prior to use. Fifty pre-treated seeds were put in each Petri dish (9 cm diameter) lined with one layer of Whatman No. 1 filter paper. An aliquot (5 ml) of each concentration of AVF was added to the Petri dish. The Petri dishes were then sealed with parafilm and maintained in a growth incubator with a diurnal temperature cycle of 25°C in light and 15°C in dark and a 12 h photoperiod. A randomized complete block design with three replicates was used. Seeds with >1 mm radical growth were considered as germinated. The germination rate, root and shoot lengths were measured after 20 days of incubation.

### Solid Phase Microextraction (SPME) Procedure

SPME is a rapid, solvent-free, low-cost technique for sample preparation and has been successfully used for the extraction of analytes from aqueous and gaseous samples [18]–[19]. In this work, the analytes in AVFs were extracted by using a manual SPME holder (Sigma–Aldrich/Bellefonte, PA, USA) before gas chromatograph–mass spectrometry (GC-MS) analysis. The fiber was conditioned prior to use by heating it at 250°C for 30 min in the GC injecting unit during a blank run. The fiber was then exposed to the AVF in 10-ml glass vials for 1 min with magnetic stirring at room temperature. After extraction, analytes on the fiber were immediately desorbed in the injector port of the gas chromatograph, separated and detected as described in the following section.

### Chemical Analysis of AVFs

The AVFs were analyzed by GC-MS with the use of J & W DB-5 fused silica capillary column (30 m×0.25 mm×0.25 µm) in a Varian 3800 gas chromatograph directly coupled to a Varian Saturn 2000 Ion Trap (ITD) mass spectrometer controlled by a Saturn GC/MS workstation (v5.2). Gas chromatography operating conditions followed those described by Adams [20]: 240°C injector and transfer line temperature; 60 to 250°C at 3°C/min oven temperature, with a final hold time of 8.67 min at 250°C (total run time 72.0 min); Helium carrier gas; Splitless. Mass spectrometry acquisition parameters were: full scan with scan range 41–415 amu; 1.0 s scan time; 1 count threshold; AGC mode on; 5 microscans; 1.8 min filament delay. Column head pressure was adjusted to 13.0 psi.

Compounds were identified by comparing their Kovats retention indices (KI), retention times and their mass fragmentation pattern with data in Adams [20], aided with NIST mass spectra library in the spectrometer database. The retention times of a homologous series of *n*-alkanes (C_8_–C_20_) were determined under the same operating conditions and used for the calculation of KI. Quantification of volatile components in AVFs was carried out by peak area normalisation measurements. The relative percentage of each component was calculated by dividing its GC-MS response (expressed as peak area) by the total peak area of chromatogram (set as 100%) of all components.

### Statistical Analysis

The dose-response data were subjected to the analysis of whole-range assessment proposed by An et al. [21]. The whole-range assessment considers overall effect/response across the whole range of application rates, instead of assessing the effect of each individual rate on test species. The program WESIA (Whole-range Evaluation of the Strength of Inhibition in Allelopathic-bioassay) developed by Liu et al. [22] was used to calculate the inhibition index based on the following equation:
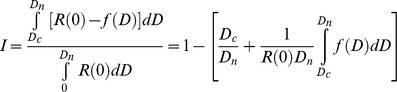
Where the *0, D_1_, D_2,_ … D_n_* are the dose-concentrations tested and the *R(0), R(D_1_), R(D_2_), … R(D_n_)* are the corresponding responses, respectively. The *D_c_* is the threshold dose at which response equals the value of control and above which the responses are inhibitory. The *f(D)* represents the response function. The inhibition index is a summary of the overall biological response of an organism to a tested allelochemical or equivalent and provides a relative strength indicator of biological response. Large values indicate that the species is sensitive or that the allelochemical possesses strong phytotoxic potential/biological activity, whilst small values indicate tolerance or weak potential/biological activity.

## Results

### AVFs on Germination of Silverleaf Nightshade

**Figure 1:**
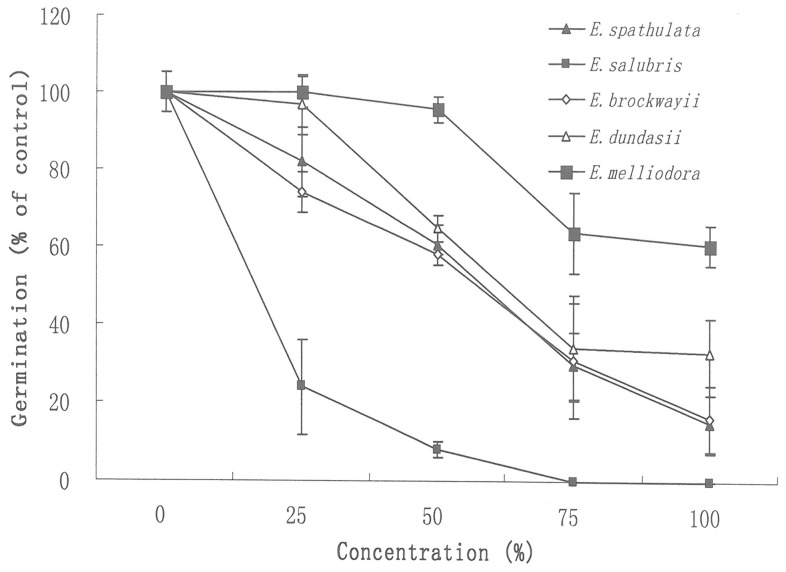
Effect of AVFs from *Eucalyptus* species on germination of silverleaf nightshade. The *Eucalyptus* species tested were *E. spathulata*, *E. salubris*, *E. dundasii*, *E. brockwayii* and *E. melliodora*. Germination of the bioassay without AVFs (water control) was taken as 100%. The germination of the bioassays with different concentrations of AVFs was then calculated relative to the water control. Error bars show standard error of the means (n = 3).

All AVFs tested inhibited the germination of silverleaf nightshade, depending on the concentration and the species (Table 1, Figure 1). The inhibition increased with the increasing concentrations. The AVFs from *E. spathulata*, *E. salubris*, *E. brockwayii* and *E. dundasii* reduced the germination rate of silverleaf nightshade by 39.8%, 92.0%, 42.0% and 35.2% respectively at a concentration of 50% and by 85.2%, 100.0%, 84.1% and 67.1% respectively at a concentration of 100%. However, the inhibition differed between species. The AVFs of the four *Eucalyptus* species selected had a higher inhibition than that of *E. melliodora* (the control species). *E. salubris* showed the inhibitoriest activity, with no germination observed at a concentration of 75% whereas the germination was reduced by only 36.4% at the same concentration of the AVF from the control species, *E. melliodora*. The inhibition potential was ranked in a decreasing order as *E. salubris*, *E. brockwayii*, *E. spathulata*, *E. dundasii* and *E. melliodora* based on the whole range assessment (Table 1).

**Table 1:** Inhibition potential of AVFs from different *Eucalyptus* species on the germination and seedling growth of silverleaf nightshade.

Species	Germination	Species	Root length	Species	Shoot length	Inhibition potential
	Inhibition Index (%)		Inhibition Index (%)		Inhibition Index (%)	
*E. salubris*	80.7	*E. salubris*	82.8	*E. salubris*	77.3	Strong
*E. brockwayii*	44.7	*E. brockwayii*	69.0	*E. spathulata*	61.0	
*E. spathulata*	42.7	*E. spathulata*	66.7	*E. brockwayii*	56.4	
*E. dundasii*	34.0	*E. dundasii*	55.2	*E. dundasii*	49.3	
*E. melliodora*	15.8	*E. melliodora*	46.6	*E. melliodora*	44.8	Weak

### AVFs on Seedling Growth of Silverleaf Nightshade

**Figure 2:**
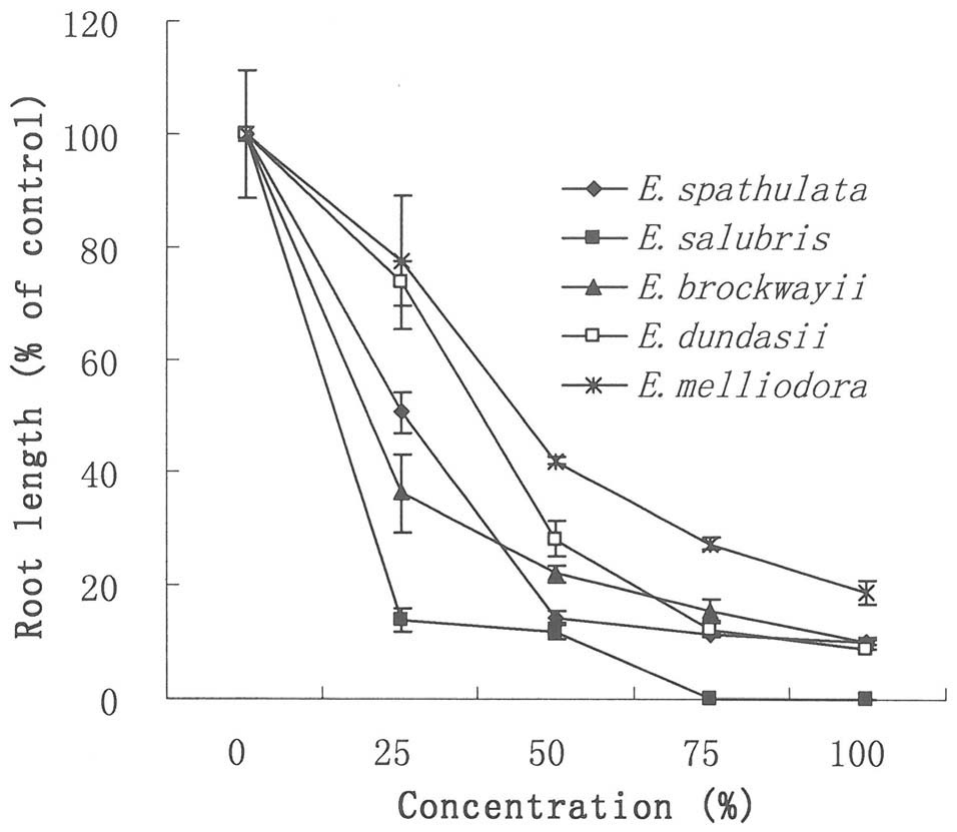
Effect of AVFs from *Eucalyptus* species on root growth of silverleaf nightshade. The *Eucalyptus* species tested were *E. spathulata*, *E. salubris*, *E. dundasii*, *E. brockwayii* and *E. melliodora*. Root length of the bioassay without AVFs (water control) was taken as 100%. The root lengths of the bioassays with different concentrations of AVFs were then calculated relative to the water control. Error bars show standard error of the means (n = 3).

It was also observed that the root length of silverleaf nightshade seedlings was decreased when exposed to the AVFs, depending on the concentration and the species (Table 1, Figure 2). The higher concentrations of AVFs used resulted in higher inhibitory effects on silverleaf nightshade. The root growth was inhibited by 85.6%, 88.2%, 78.0% and 71.9% respectively at a concentration of 50% and by 90.2%, 100.0%, 90.1% and 91.1% respectively at a concentration of 100% for AVFs from *E. spathulata*, *E. salubris*, *E. brockwayii* and *E. dundasii*. The AVF of *E. salubris* demonstrated the highest inhibition on root growth, whereas the AVF of *E. melliodora* was the least inhibitory. The inhibition potential was ranked in a decreasing order similar to the germination inhibition reported above.

**Figure 3:**
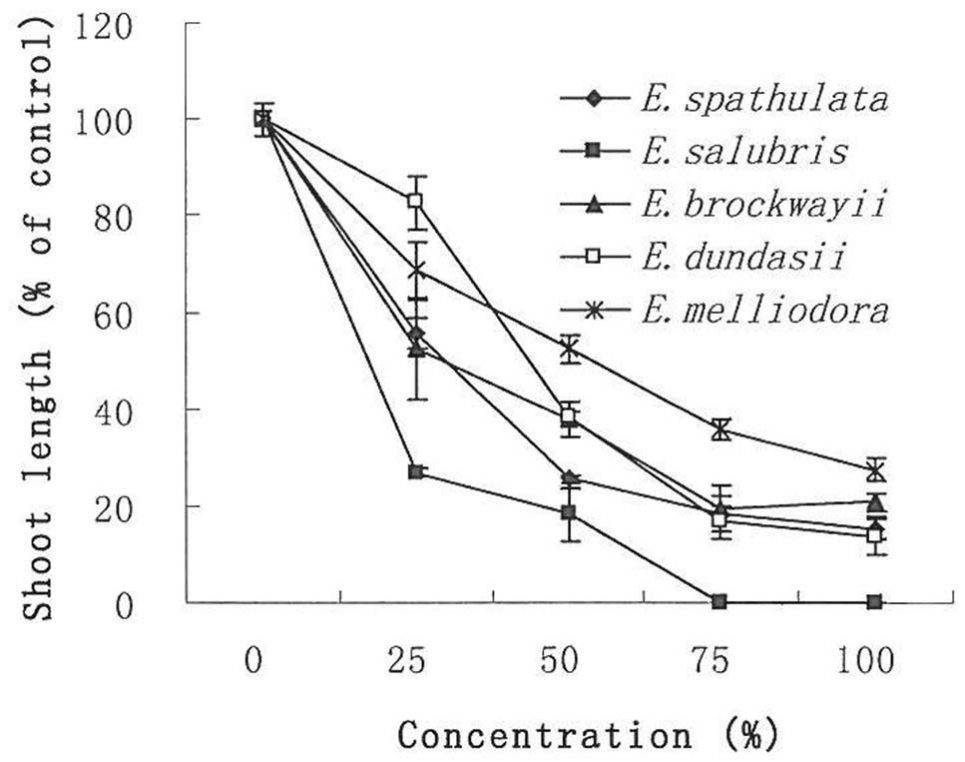
Effect of AVFs from *Eucalyptus* species on shoot growth of silverleaf nightshade. The *Eucalyptus* species tested were *E. spathulata*, *E. salubris*, *E. dundasii*, *E. brockwayii* and *E. melliodora*. Shoot length of the bioassay without AVFs (water control) was taken as 100%. The shoot lengths of the bioassays with different concentrations of AVFs were then calculated relative to the water control. Error bars show standard error of the means (n = 3).

The AVFs also significantly suppressed the shoot growth of silverleaf nightshade seedlings (Table 1, Figure 3). This inhibition became more severe with increased dose used. The application of the AVF of *E. salubris* resulted in 73.1% inhibition at a concentration of 25% and 100% inhibition when the concentration was 75%. Different degrees of inhibition were again observed between species. The inhibition potential was ranked in a decreasing order as *E. salubris*, *E. spathulata*, *E. brockwayii*, *E. dundasii* and *E. melliodora*. However, the inhibition index was lower than that in root growth for all species (Table 1), indicating that the root growth of silverleaf nightshade was more sensitive to AVFs than shoot growth.

### Chemical Analysis of AVFs by GC-MS

**Figure 4:**
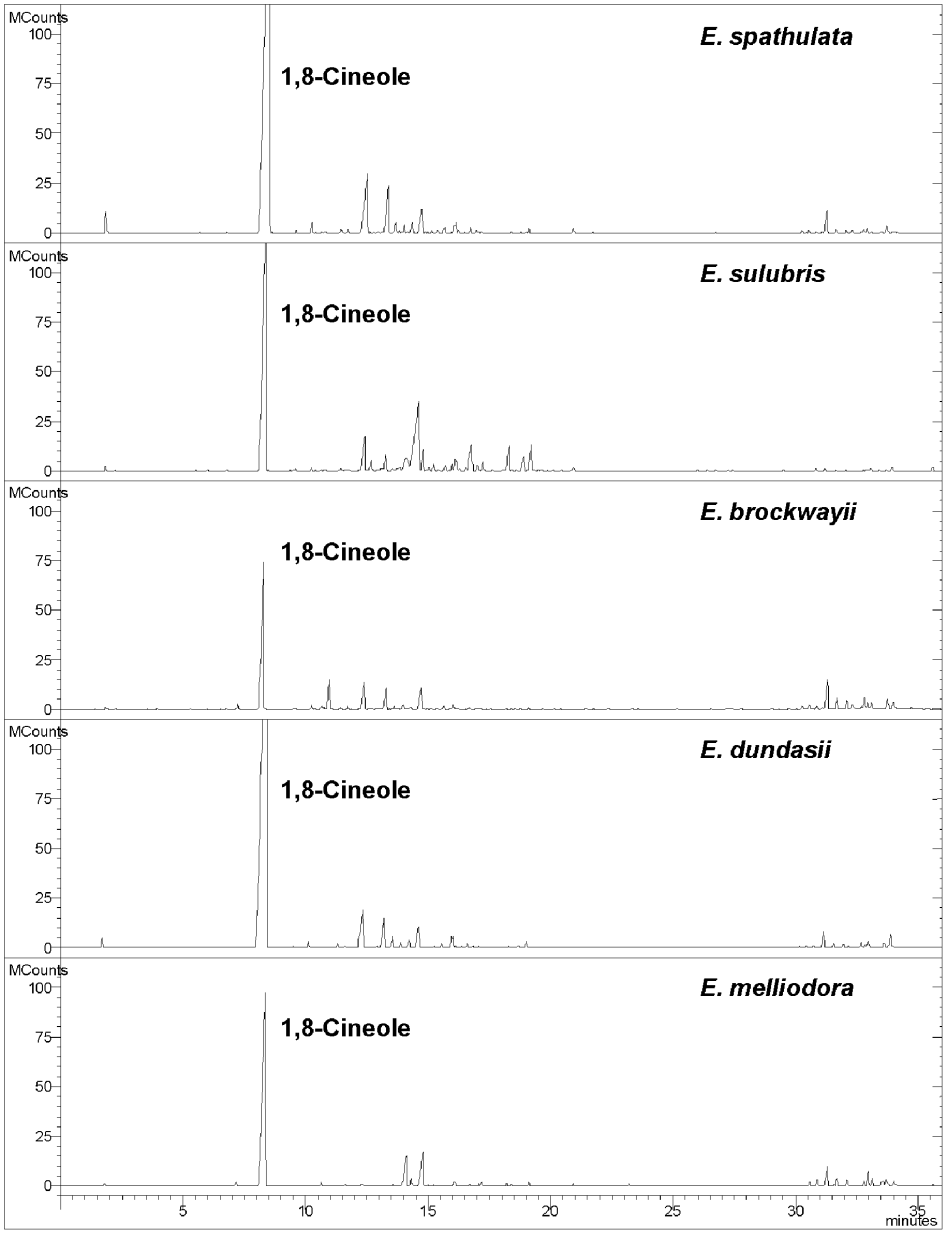
GC-MS profile of AVFs from different *Eucalyptus* species.

The AVF composition from the four eucalyptus leaves was analyzed by GC-MS. The results were presented in Table 2 and Figure 4. There were 32 compounds identified in the AVF from *E. spathulata* leaves. It was dominated by 1,8-cineole (74.0%), pinocarvone (3.8%), *trans*-pinocarveol (7.2%), *alpha*-terpineol (2.5%) and globulol (1.4%). A total of 29 compounds were identified in the AVF of *E. salubris*. The main components were 1,8-cineole (47.5%), isomenthol (15.9%), pinocarvone (1.5%), *trans*-pinocarveol (4.7%) and *alpha*-terpineol (1.9%). Thirty-five compounds in the AVF of *E. brockwayii* were identified, with the predominant compounds being 1,8-cineole (37.1%), isopentyl isovalerate (5.9%), pinocarvone (4.2%), *trans*-pinocarveol (7.0%), *alpha*-terpineol (5.8%) and globulol (6.9%). GC–MS analyses also led to the identification of 34 different compounds in AVF of *E. dundasii*, with 1,8-cineole (80.1%), pinocarvone (2.2%), *trans*-pinocarveol (4.3%), *alpha*-terpineol (2.1%) and globulol (1.0%) as the main components.

**Table 2:** Main compounds identified in AVFs and their relative percentage.

Compounds/Species	*E. spathulata*	*E. salubris*	*E. brockwayii*	*E. dundasii*	*E. melliodora*
**1,8-Cineole**	74.0	47.5	37.1	80.1	54.7
**Isopentyl isovalerate**	--	--	5.9	--	--
**Isomenthol**	--	15.9	0.5	--	1.2
**Pinocarvone**	3.8	1.5	4.2	2.2	--
***trans*** **-Pinocarveol**	7.2	4.7	7.0	4.3	--
***alpha*** **-Terpineol**	2.5	1.9	5.8	2.1	9.6
**Globulol**	1.4	--	6.9	1.0	3.4
**Other main compounds**	Santolinyl acetate (0.6), 6-camphenone (0.5)	Cumin aldehyde (3.1), terpin-4-ol (3.0), carvacrol (2.7), dihydro-linalool acetate (2.4), thymol (1.6), piperitone (0.8), *trans*-carveol (0.5), santolinyl acetate (0.5), neo-iso-dihydro carveol (0.5)	Viridiflorol (2.3), *alpha*-eudesmol (1.8), *beta*-acorenol (1.5), *gamma*-eudesmol (1.3), phellandrene (1.0), neo-iso-dihydro carveol (1.0), spathulenol (0.6), *trans*-carveol (0.6), terpin-4-ol (0.6), 6-camphenone (0.6), linalool (0.5)	*alpha*-Eudesmol (1.2), neo-iso-dihydro carveol (0.7), santolinyl acetate (0.7)	Terpin-4-ol (6.9), *gamma*-eudesmol (2.2), *alpha*-muurolol (2.0), spathulenol (1.1), *cis*-arteannuic alcohol (1.1), *beta*-acorenol (1.0), neo-iso-dihydro carveol (0.9), *alpha*-eudesmol (0.8), himachalol (0.7)

## Discussion

The bioassay showed that all AVFs tested had differential inhibition on the germination and seedling growth of silverleaf nightshade. The AVFs from *E. spathulata*, *E. salubris*, *E. brockwayii* and *E. dundasii* had stronger phytotoxic effect on silverleaf nightshade when compared with the control species *E. melliodora*. Among these four selected species, *E. salubris* had the highest inhibition index for germination, root and shoot growth of silverleaf nightshade. These results confirmed that the AVFs from *Eucalyptus* species had phytotoxicity on weed plant.

The identification and quantification of AVFs revealed that 1,8-cineole was the most abundant components in the AVFs of all *Eucalyptus* species tested. 1,8-Cineole has been confirmed to have many bioactivities [1], [23]. The herbicidal activity of 1,8-cineole has been tested on a wide range of weed species, including *E. crus-galli*, *Cassia obtusifolia* [24], *Ageratum conyzoides* L. [25] and *Amaranthus viridis* [26]. It has been successfully used as a lead compound in the development of an active grass herbicide for use in broadleaf crops such as soybeans [*Glycine max* (L.) Merr.] [27]. Therefore, 1.8-cineole, as the most dominant component in AVFs, may account for the phytotoxicity observed. However, the % 1,8-cineole in the AVFs did not fully explain the differences in phytotoxicities between the species. As showed in Tables 1–2 and Figure 4, the relative percentage (37.1%) and GC-MS response in terms of the peak area for 1,8-cineole in AVF of *E. brockwayii* was the lowest among the four *Eucalyptus* species selected, whereas the inhibition potential on silverleaf nightshade was moderate and even higher than that of *E. dundasii* with the highest relative percentage (80.1%) of 1,8-cineole. These results indicated that 1,8-cineole was not the most potent compound for the observed biological activities of AVFs. Other compounds including minor components could also contribute to the overall suppression of AVFs. In fact, many other individual compounds identified in eucalyptus oils, such as *alpha*-terpineol, citronellal, citronellol and *alpha*-pinene, have been confirmed to have phytotoxic activity [1], [28]. The chemical composition of AVFs varied between species. The combined effects of these compounds and the difference in chemical composition between *Eucalyptus* species may be used to explain the difference in their phytotoxicities against silverleaf nightshade. Further research on the phytotoxicities of AVFs and individual compounds from these *Eucalyptus* species on more weed plants will improve our understanding the relation between their bioactivity and chemical composition.

It was further found from Table 2 that the main compounds in AVFs were also present in the corresponding essential oils identified previously [15]. These results indicated that both extracts from eucalyptus leaves contained aqueous and phytotoxic compounds, which could be easily leached onto the ground. However, further investigation under field conditions is needed to determine the concentration of these bioactive compounds in the soil.

## Conclusions

The results obtained in this study indicated that the AVFs from the selected *Eucalyptus* species had strong phytotoxic effects on the germination and seedling growth of silverleaf nightshade. The chemical analysis showed that the main components of the AVFs were 1,8-cineole, isopentyl isovalerate, isomenthol, pinocarvone, *trans*-pinocarveol, *alpha*-terpineol and globulol, depending on species. The AVF inhibition between species was different on the weed, which could be due to the joint effects of compounds present in the AVFs as these compounds were present in different quantities and ratio between *Eucalyptus* species.
